# Restorative resection in robot-assisted *versus* laparoscopic total mesorectal excision: systematic review and meta-analysis

**DOI:** 10.1093/bjs/znag106

**Published:** 2026-10-06

**Authors:** Charlotte M S Genders, Jory Wentink, Ritch T J Geitenbeek, Roel Hompes, Jasper B van Praagh, Esther C J Consten

**Affiliations:** Department of Surgery, University Medical Centre Groningen, Groningen, The Netherlands; Department of Surgery, Meander Medical Centre, Amersfoort, The Netherlands; Department of Surgery, University Medical Centre Groningen, Groningen, The Netherlands; Department of Surgery, Meander Medical Centre, Amersfoort, The Netherlands; Department of Surgery, Meander Medical Centre, Amersfoort, The Netherlands; Department of Surgery, Amsterdam University Medical Centre, Amsterdam, The Netherlands; Cancer Treatment and Quality of Life, Cancer Centre Amsterdam, Amsterdam, The Netherlands; Department of Surgery, University Medical Centre Groningen, Groningen, The Netherlands; Department of Surgery, Deventer Hospital, Deventer, The Netherlands; Department of Surgery, University Medical Centre Groningen, Groningen, The Netherlands; Department of Surgery, Meander Medical Centre, Amersfoort, The Netherlands

## Abstract

**Background:**

The surgical treatment of rectal cancer is based on the principle of total mesorectal excision (TME). Owing to its technological advantages, robot-assisted total mesorectal excision (R-TME) is hypothesized to facilitate primary anastomosis more frequently than laparoscopic total mesorectal excision (L-TME). This systematic review and meta-analysis aimed to investigate whether R-TME is associated with a higher likelihood of restorative surgery compared with L-TME.

**Methods:**

This systematic review and meta-analysis was conducted in accordance with PRISMA guidelines. The protocol was prospectively registered in PROSPERO, the international prospective register of systematic reviews (registration number: 1106767). PubMed/MEDLINE, Embase, the Web of Science, and the Cochrane Library were searched for studies from January 2010 to January 2026. Pooled ORs and mean differences were calculated using a random-effects model.

**Results:**

Twenty studies were included, reporting on 7133 patients. The pooled analysis demonstrated a significantly higher likelihood of restorative surgery with R-TME compared with L-TME (OR 1.43 (95% c.i. 1.04 to 1.98)). In a subgroup analysis of studies including patients after 2020, a stronger effect was observed (OR 2.86 (95% c.i. 1.80 to 4.53)). No significant differences were observed in anastomotic leakage, postoperative complications, reoperations, and readmissions. R-TME was associated with a lower conversion rate compared with L-TME (OR 0.37 (95% c.i. 0.28 to 0.49)).

**Conclusion:**

R-TME is associated with a higher likelihood of primary anastomosis formation while maintaining a comparable safety profile. The more pronounced effect in recent studies likely reflects increasing surgical expertise. Future research should determine whether these advantages translate into measurable improvements in patient quality of life.

## Teaser

Robot-assisted total mesorectal excision is associated with a higher likelihood of primary anastomosis and a lower conversion rate compared with laparoscopic surgery, without increasing postoperative morbidity. This association is more pronounced in recent studies, likely reflecting increased expertise with robot-assisted surgery.

## Introduction

Rectal cancer remains a major global health burden and is increasingly diagnosed in many regions of the world^1^. Surgery is the cornerstone of curative treatment and is based on the principle of total mesorectal excision (TME), introduced by Heald *et al*.^2^ in 1982, which standardized oncological rectal surgery and improved local control. Over the past decades, minimally invasive techniques have been widely adopted for TME, most notably robot-assisted total mesorectal excision (R-TME) and laparoscopic total mesorectal excision (L-TME).

Several studies have compared robot-assisted and laparoscopic approaches for rectal cancer. A recent multicentre RCT by the REAL study group reported superior oncological resection quality and improved postoperative recovery with robotic surgery compared with conventional laparoscopy^3,4^. Likewise, the COMPARE study found that R-TME was associated with significantly lower risks of conversion, perioperative blood transfusion, and postoperative complications, as well as a shorter length of hospital stay^5^. Nevertheless, current guidelines still consider both robotic and laparoscopic techniques as standard minimally invasive approaches, as many previous studies have not demonstrated clinically relevant differences in key surgical or oncological outcomes when procedures are performed by experienced surgeons^6^.

Beyond short-term perioperative outcomes, the feasibility of constructing a primary anastomosis is a critical consideration in rectal cancer surgery. When sphincter preservation is feasible, a restorative low anterior resection (RLAR) with primary anastomosis is generally preferred. In patients at high risk of morbidity or mortality from anastomotic leakage (AL), or when poor functional outcomes are anticipated, a non-restorative low anterior resection (NRLAR) or abdominoperineal resection (APR) may be chosen, both resulting in a permanent colostomy. Restorative surgery therefore has major implications for long-term function and quality of life. It has been suggested that R-TME facilitates primary anastomosis formation more frequently than L-TME, owing to enhanced dexterity, improved ergonomics, and stable three-dimensional visualization of the pelvic anatomy^7,8^. These technical advantages may reduce the need for non-restorative resections and permanent stomas.

However, the magnitude of any benefit of R-TME on restorative procedure rates remains uncertain. Existing studies that report restorative surgery rates were not primarily designed or powered to evaluate this outcome. To the authors’ knowledge, no systematic review has specifically focused on restorative surgery in minimally invasive TME for rectal cancer. Therefore, a systematic review and meta-analysis was performed to determine whether R-TME is associated with a higher rate of restorative surgery compared with L-TME.

## Methods

This systematic review was conducted and written in accordance with the PRISMA guidelines^9^. The protocol was prospectively registered in PROSPERO, the international prospective register of systematic reviews (registration number: 1106767).

### Eligibility criteria

The study eligibility criteria were defined in line with the Population, Intervention, Comparison, Outcome, and Study design (PICOS) framework. Studies were deemed eligible if they met all of the following criteria: included adult patients (aged ≥18 years) with rectal cancer; compared R-TME with L-TME; reported the proportion of patients undergoing restorative surgery, either as a pre-specified outcome or as a reported surgical endpoint; and used an RCT or prospective cohort design.

Studies were excluded if they met any of the following criteria: publication language other than English, Dutch, French, German, or Spanish; not primary research, including, amongst others, reviews, (conference) abstracts, commentaries, and editorials; sample size less than ten patients; full text unavailable; or published before 2010, due to the significant advances made in rectal surgery and robotic platforms over the past decade. In case of overlapping study populations, the study with the largest sample size and longest follow-up duration was included.

### Literature search and study selection

A comprehensive systematic search was independently conducted by two researchers across PubMed/MEDLINE, EMBASE, the Web of Science, and the Cochrane Library up to 1 January 2026. The literature search was conducted using a predefined search string (*Appendix S1*), which was critically appraised and refined by a professional medical librarian.

Two independent researchers (C.M.S.G. and J.W.) screened titles and abstracts using Rayyan, a web-based tool for systematic review management^10^. The full text of articles deemed potentially relevant was then assessed by the same reviewers to determine definite inclusion. Reasons for exclusion were documented at the full-text stage. Discrepancies were resolved through discussion and, when necessary, consultation with three additional authors (R.T.J.G., J.B.v.P., and E.C.J.C.). Reference lists of all the included studies were reviewed to identify potentially relevant articles. Systematic reviews were excluded, but reference lists were screened for additional articles.

### Data collection

Data were extracted from the included studies using a standardized extraction form by two independent reviewers (C.M.S.G. and J.W.). The primary outcome was the restorative surgery rate, defined as the proportion of patients undergoing a restorative procedure with primary anastomosis irrespective of whether a defunctioning stoma was simultaneously constructed. Secondary outcomes included operating time, conversion to open surgery, postoperative complications, AL, reoperations, and readmissions.

For each eligible study the following data were extracted: study characteristics (first author, year of publication, journal, country, study design, funding, and conflicts of interest); baseline characteristics of the study population (number of patients, sex, age, BMI, and ASA grade^11^); preoperative data (TNM stage, neoadjuvant therapy (NAT), and tumour height, i.e. distance to the anorectal junction); operative data (surgeon experience, surgical technique, type of procedure, and stoma creation); and postoperative data (AL and AL definition, Clavien–Dindo grade, and length of hospital stay)^12^. Different reporting outcomes were evaluated for inclusion on a case-by-case basis.

### Quality assessment

The methodological quality and risk of bias of the included studies were independently assessed by two authors (C.M.S.G. and J.W.) using the Cochrane Risk of Bias 2 (RoB 2) tool for RCTs and the methodological index for non-randomized studies (MINORS) tool for prospective non-randomized studies^13,14^. The Grading of Recommendations Assessment, Development, and Evaluation (GRADE) working group approach was applied to evaluate the quality of the cumulative evidence for the primary outcome^15^. Disagreements in quality assessment were resolved through discussion, in which three additional authors were involved (R.T.J.G., J.B.v.P., and E.C.J.C.).

### Data analysis

Statistical analysis was performed using R statistical software (version 4.5.1)^16^. Outcomes reported in three or more studies were included in the meta-analysis. For binary outcomes, pooled ORs with 95% confidence intervals were calculated. For continuous outcomes, mean differences (MDs) were calculated.

Statistical heterogeneity was assessed using Cochran’s Q test (with *P* < 0.050 indicating significant heterogeneity) and quantified using both *I*^2^ and τ^2^. Where τ^2^ indicated meaningful between-study variance, random-effects models using the DerSimonian and Laird method were applied. Where τ^2^ was zero or negligible, a common-effect model using the Mantel–Haenszel method was applied. Publication bias and small-study effects were evaluated by visual inspection of funnel plots and by Egger’s regression test for outcomes reported by at least ten studies. Sensitivity analyses were conducted to explore the robustness of the findings and potential sources of heterogeneity, stratifying by study interval, study size, study design, proportion of male patients, and use of NAT. A two-sided *P* value <0.050 was considered statistically significant.

## Results

### Study selection

The literature search yielded 3753 unique records after duplicates were removed (*Fig. 1*^17^). After screening titles and abstracts, 105 articles were selected for full-text assessment, resulting in the inclusion of 20 studies in the quantitative analysis. The characteristics of the included studies are detailed in *Table 1*. The included studies, published between 2010 and 2026, encompassed a total of 7133.4 patients. One study applied propensity score weighting, resulting in an effective sample size of 7133.4 rather than an integer. *Tables S1–S3* present patient demographics, surgeon experience, perioperative outcomes, and postoperative outcomes for the included studies.

**Figure 1:**
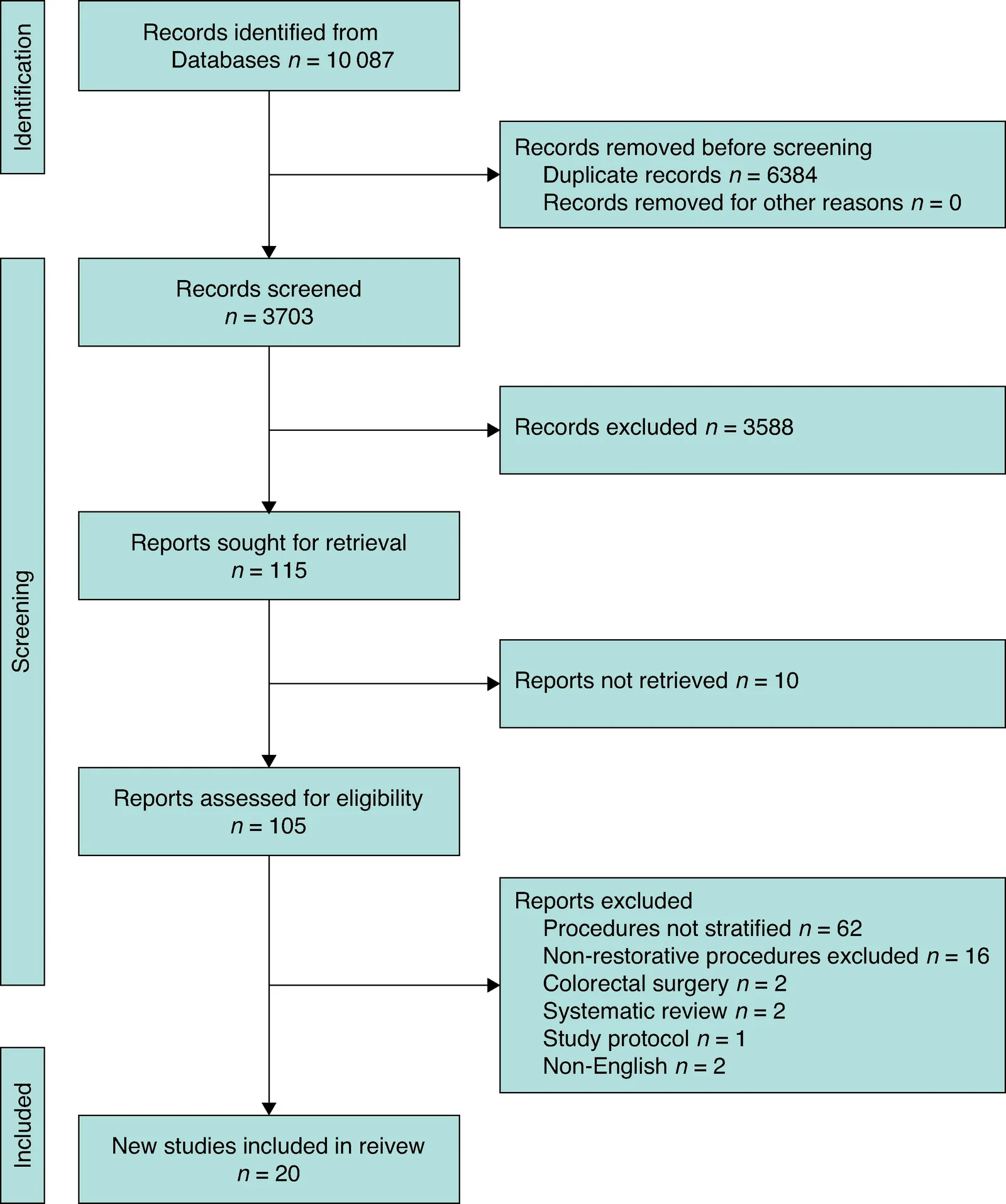
Flow chart depicting study selection process

**Table 1:** Characteristics of the included studies

Study	Country	Study design	Study interval	Primary outcome	Patients, *n*	Restorative R-TME	Restorative L-TME
Bianchi et al.^18^	Italy	NRPS	March 2008–June 2009	Short-term oncological safety	50	18 (72)	19 (76)
Kim et al.^19^	South Korea	NRPS	June 2009–November 2009	Urogenital function	69	29 (97.7)	38 (97)
Park et al.^20^	Korea	Retrospective analysis of NRPS	February 2009–December 2010	Urinary and sexual function	64	31 (97)	31 (97)
Levic et al.^21^	Denmark	Retrospective analysis of NRPS	2010–2012	Short-term oncological outcomes	92	32 (57)	20 (56)
Alleman et al.^22^	Switzerland	Retrospective analysis of NRPS	May 2012–January 2014	TME quality	60	18 (90)	34 (85)
Yamaguchi et al.^23^	Japan	Retrospective analysis of NRPS	April 2010–April 2015	Short-term operative outcomes	442	168 (93.9)	195 (89.9)
Ahmed et al.^24^	UK	Retrospective analysis of NRPS	May 2005–November 2015	Perioperative outcomes	184	85 (87)	63 (77)
Jayne et al.^25^	UK	RCT	January 2011–September 2014	Conversion	471	187 (77.6)	184 (80.3)
Law et al.^26^	Hong Kong	NRPS	January 2008–June 2015	Conversion and postoperative complications	391	206 (93.6)	152 (87.4)
Panteleimonitis et al.^27^	UK	Retrospective analysis of NRPS	December 2006–September 2014	Short-term mortality and morbidity	126	40 (83)	63 (81)
Crolla et al.^28^	Netherlands	Retrospective analysis of NRPS	2005–2015	Conversion and complications	352	112 (66.7)	122 (66.3)
Kim et al.^29^	South Korea	RCT	February 2012–March 2015	Short-term oncological outcomes and urinary function	139	65 (99)	70 (96)
Megevand et al.^30^	Italy	Retrospective analysis of NRPS	January 2011–December 2015	Short-term postoperative outcomes	70	28 (80)	30 (85.7)
Tolstrup et al.^31^	Denmark	RCT	November 2012–April 2014	Perioperative pain	51	13 (59)	16 (64)
Feng et al.^7^	China	RCT	July 2016–December 2020	Three-year locoregional recurrence	1171	486 (82.3)	449 (76.8)
Liu et al.^32^	China	NRPS	January 2017–January 2020	Urogenital function	135	87 (97)	36 (80)
Myrseth et al.^33^	Norway	Retrospective analysis of NRPS	January 2014–December 2018	Conversion	1284	191 (50.9)	552 (60.7)
Duhoky et al.^7^	UK	Retrospective analysis of NRPS	January 2014–January 2024	Length of stay, operating time, and conversion	1738.4*	974 (92.1)	546 (80.1)
Guerrero-Ortiz et al.^34^	Spain	NRPS	January 2022–December 2022	Cost-effectiveness	182	88 (93)	64 (74)
Kitaguchi et al.^35^	Japan	Retrospective analysis of NRPS	January 2017–March 2024	Short-term postoperative outcomes	62	15 (71)	27 (66)

Values are *n* (%) unless otherwise indicated. R-TME, robot-assisted total mesorectal excision; L-TME, laparoscopic total mesorectal excision; NRPS, non-randomized prospective study; TME, total mesorectal excision. *Propensity score weighting was used; the effective sample size was calculated as 1738.4.

### Quality of evidence

Four studies were RCTs, three of which were judged to have a low risk of bias according to the RoB 2 tool^4,25,29^ (*Fig. S1*). The remaining 16 studies were prospective cohort studies; based on the MINORS criteria, 14 were rated as having moderate and two as having good methodological quality^20,32^ (*Fig. S2*).

### Restorative surgery rates

The pooled analysis demonstrated a significantly higher likelihood of restorative surgery in the robotic group compared with the laparoscopic group, with a pooled OR of 1.43 (95% c.i. 1.04 to 1.98) (*P* < 0.029) (*Fig. 2*). In absolute terms, restorative surgery was performed in 2873 of 3464 R-TME patients (82.9%) compared with 2710 of 3617 L-TME patients (74.9%). Substantial heterogeneity was observed among the included studies (*I*^2^ = 77.3%, τ^2^ = 0.31). The 95% prediction interval ranged from OR 0.42 to 4.85, suggesting that, while the average effect favours R-TME, the benefit is not expected to be uniform across all clinical settings. Egger’s regression test did not indicate evidence of small-study effects or publication bias (t = 0.66, *P* = 0.52). Visual inspection of the funnel plot did not indicate asymmetry, supporting the absence of publication bias (*Fig. S3*). The certainty of evidence according to GRADE for primary anastomosis was rated as moderate.

**Figure 2:**
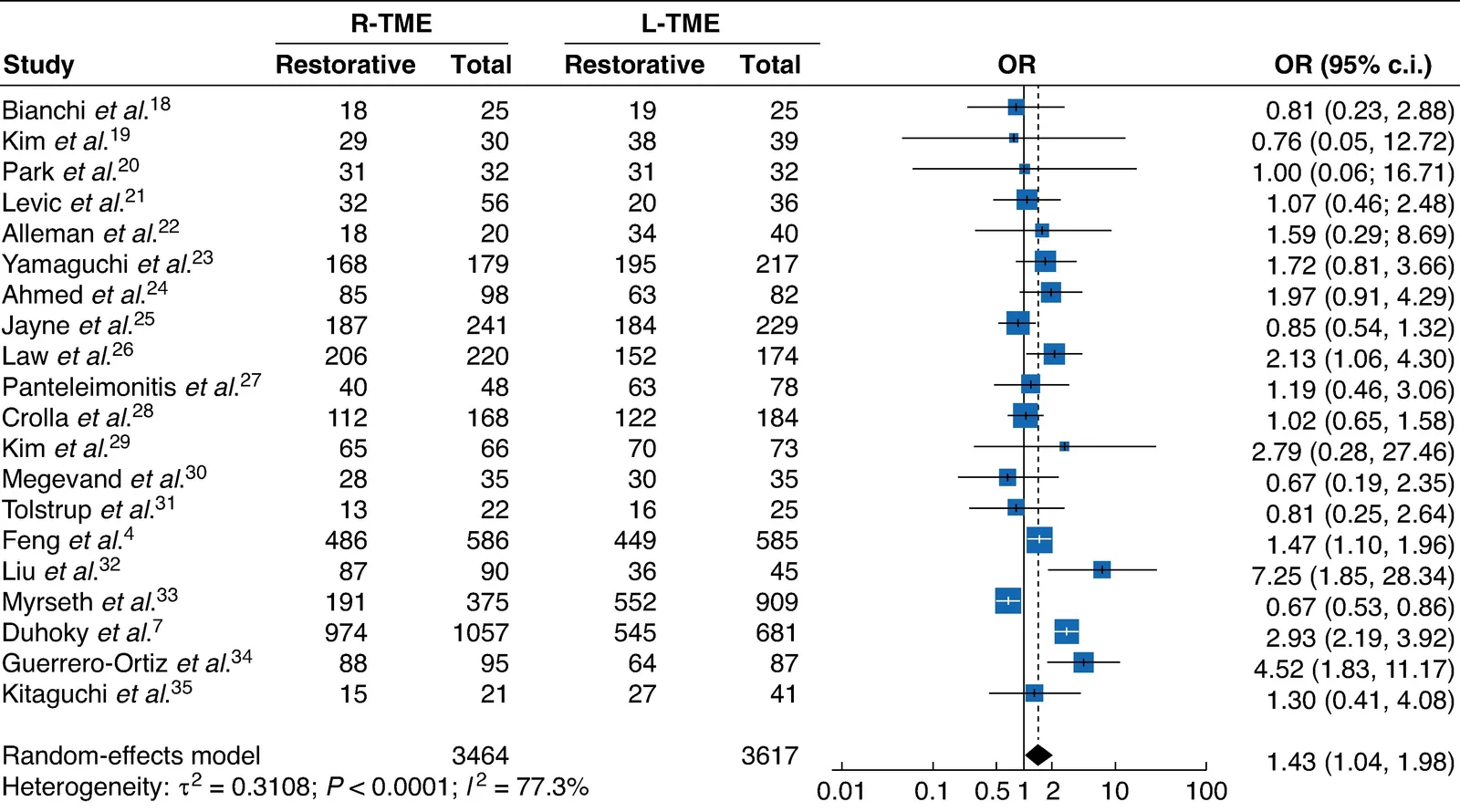
Forest plots of pooled ORs with 95% confidence intervals for restorative surgery R-TME, robot-assisted total mesorectal excision; L-TME, laparoscopic total mesorectal excision.

To explore potential sources of heterogeneity, subgroup analyses were performed. Subgroup analysis by study interval revealed a greater effect of robotic surgery in studies with an inclusion interval extending beyond 2020 (OR 2.86 (95% c.i. 1.80 to 4.53), *P* < 0.001), with moderate heterogeneity (*I*^2^ = 29.1%, τ^2^ = 0.06) (*Fig. 3*). In the subgroup of studies including patients beyond 2020, the absolute restorative surgery rates were 1077 of 1173 (91.8%) for R-TME and 636 of 809 (78.6%) for L-TME. The subgroup difference was statistically significant (*P* = 0.002). No significant subgroup differences were identified when stratifying by study size, study design (RCT *versus* prospective cohort studies), the use of NAT, the proportion of male patients, or ASA grade (*Fig. S4*). Subgroup analysis by tumour height, BMI, margin status, or clinical T-stage could not be performed due to heterogeneous and incomplete reporting across studies.

**Figure 3:**
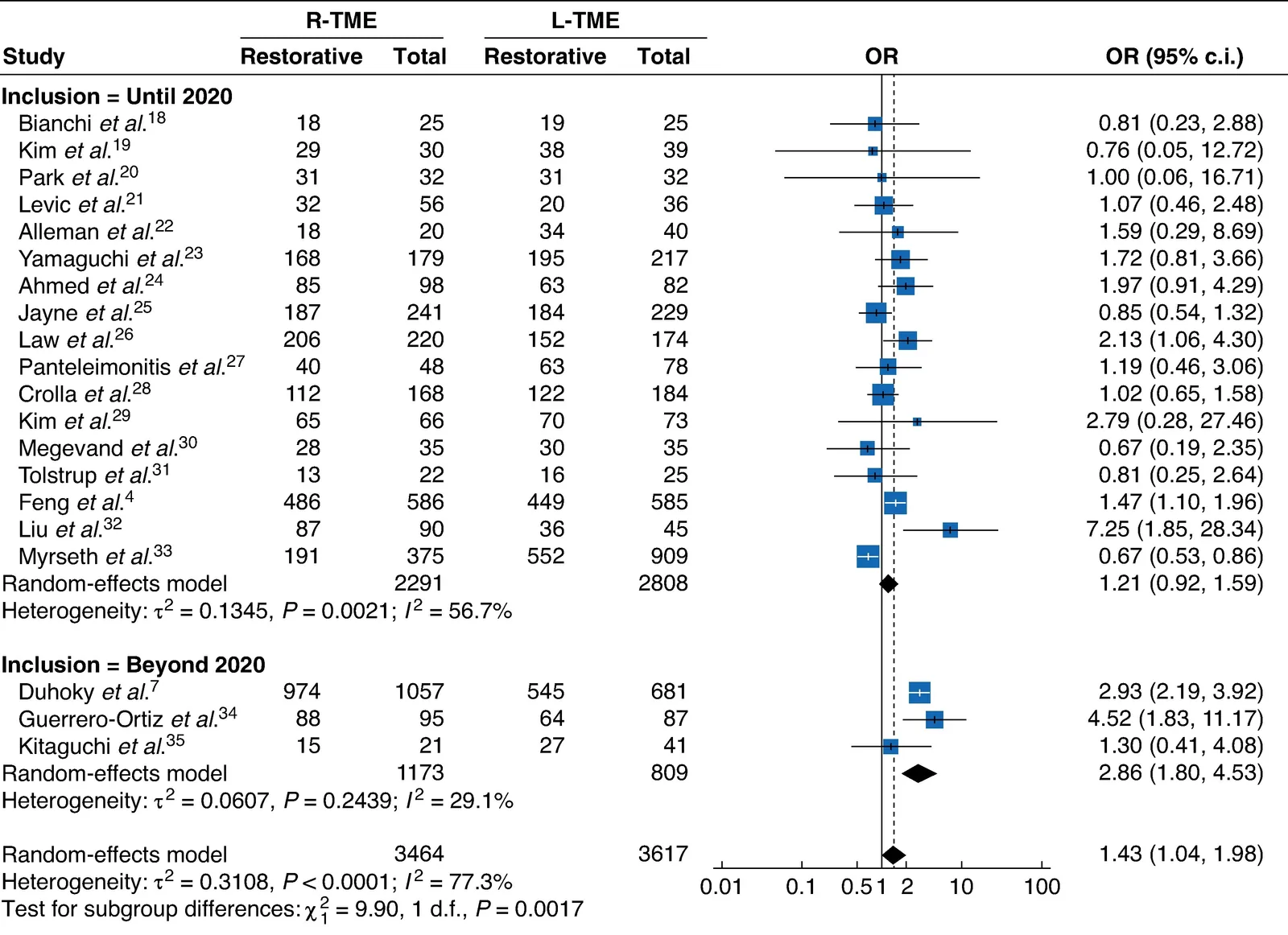
Forest plots of pooled ORs with 95% confidence intervals for subgroup analysis of inclusion interval R-TME, robot-assisted total mesorectal excision; L-TME, laparoscopic total mesorectal excision.

### Perioperative outcomes

R-TME was associated with a significantly lower conversion rate compared with L-TME (OR 0.37 (95% c.i. 0.28 to 0.49), *P* < 0.001, *I*^2^ = 30.9%, τ^2^ = 0.16). Absolute conversion rates were 87 of 2785 (3.1%) for R-TME and 254 of 2588 (9.8%) for L-TME. The operating time was significantly longer in the R-TME group (MD 36.03 (95% c.i. 8.93 to 63.13) min, *P* < 0.009, *I*^2^ = 93.6%, τ^2^ = 1044.60) (*Fig. 4*).

**Figure 4:**
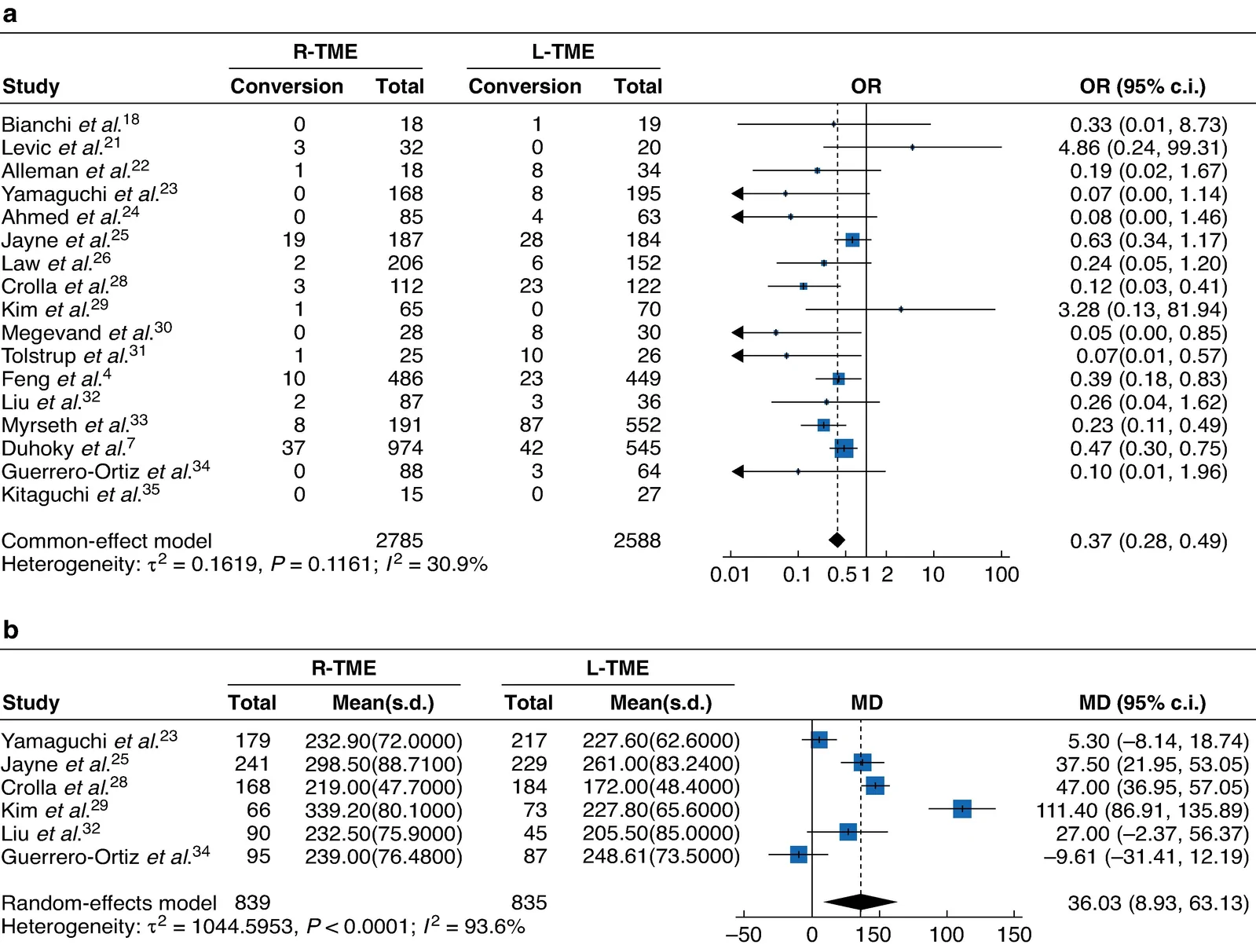
Forest plots of pooled ORs with 95% confidence intervals for perioperative outcomes **a** Conversion. **b** Operating time (min). R-TME, robot-assisted total mesorectal excision; L-TME, laparoscopic total mesorectal excision; MD, mean difference.

### Postoperative outcomes

AL was reported in 17 studies, comprising 2831 patients in the R-TME group and 2656 in the L-TME group. A standardized definition for AL was provided across 3 of the 17 studies (*Table S3*). The pooled analysis showed a non-significant trend towards a lower risk of AL in the robotic group (OR 0.72 (95% c.i. 0.45 to 1.15), *P* = 0.166) (*Fig. 5*), with substantial heterogeneity (*I*^2^ = 69.3%, τ^2^ = 0.54). Egger’s regression test found no evidence of small-study effects or publication bias (t = 1.60, *P* = 0.13). Visual inspection of the funnel plots for AL and restorative resection showed no asymmetry, supporting the absence of publication bias (*Fig. S3*). No significant differences were observed between R-TME and L-TME with regard to reoperations (OR 0.93 (95% c.i. 0.74 to 1.16), *P* = 0.508, *I*^2^ = 0%, τ^2^ = 0), readmissions (OR 0.76 (95% c.i. 0.48 to 1.20), *P* = 0.247, *I*^2^ = 42.6%, τ^2^ = 0.12), or postoperative complications (OR 0.94 (95% c.i. 0.82 to 1.08), *P* = 0.408, *I*^2^ = 33.4%, τ^2^ = 0.15).

**Figure 5:**
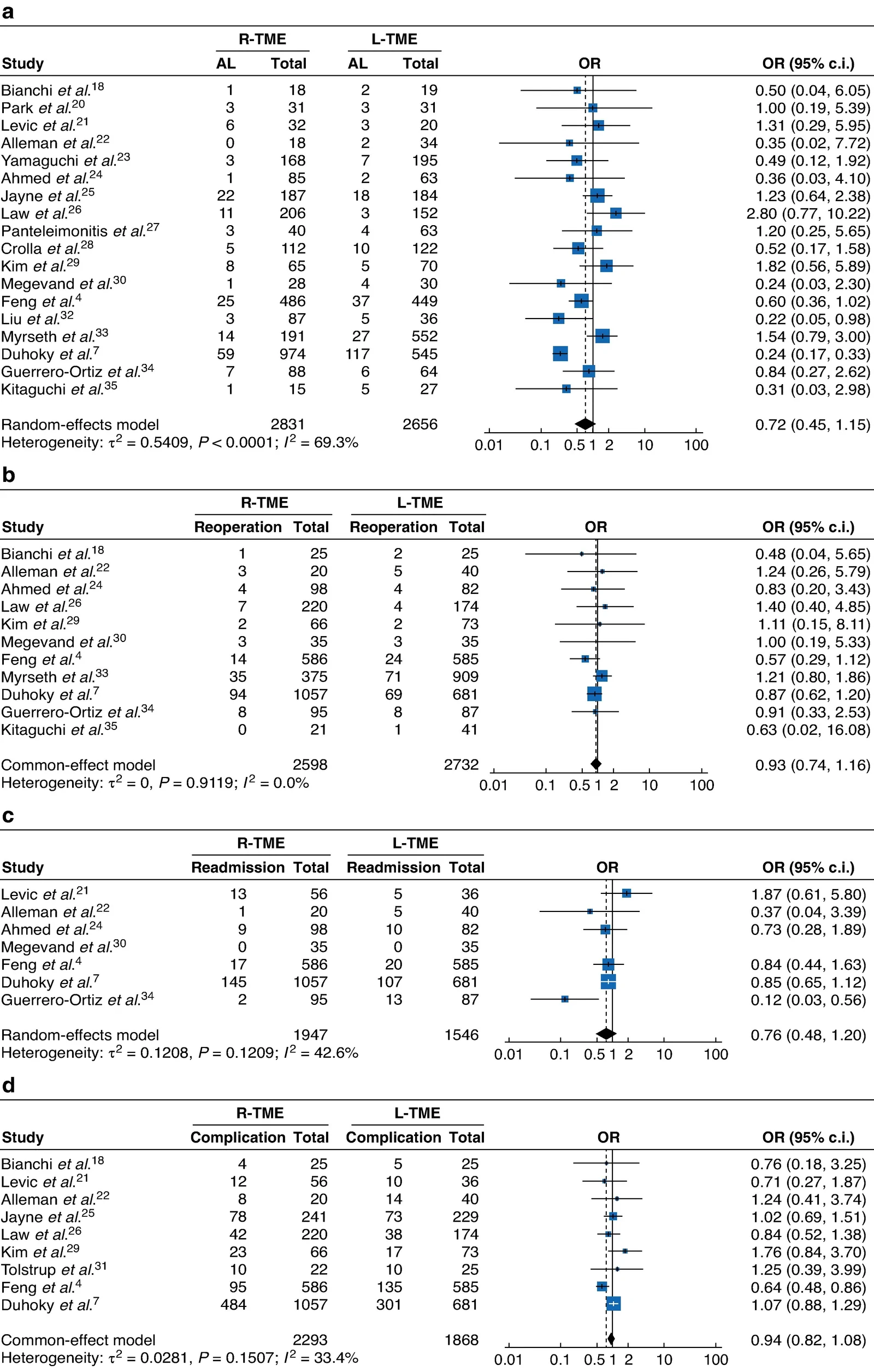
Forest plots of pooled ORs with 95% confidence intervals for postoperative outcomes **a** AL. **b** Reoperation. **c** Readmission. **d** Postoperative complication. R-TME, robot-assisted total mesorectal excision; L-TME, laparoscopic total mesorectal excision; AL, anastomotic leakage.

## Discussion

This systematic review and meta-analysis of 20 studies including >7000 patients found that R-TME is associated with a higher likelihood of restorative surgery compared with L-TME (OR 1.43 (95% c.i. 1.04 to 1.98), *P* < 0.029). Importantly, this increase in restorative procedures did not come at the expense of an increased number of ALs, overall postoperative complications, reoperations, or readmissions.

These findings have clinically relevant implications for both quality of life and oncological outcomes. The formation of a primary anastomosis avoids the need for a permanent stoma, which has been consistently associated with better health-related quality of life^36^. Furthermore, non-restorative resections are associated with worse long-term survival and oncological outcomes compared with restorative procedures, although these observations may in part reflect differences in patient selection^37,38^. The improved access, enhanced precision, and stable three-dimensional visualization provided by the robotic platform may help to overcome the challenges of adequately dissecting low rectal tumours and facilitate the formation of a primary anastomosis in a broader patient population. Nevertheless, higher restorative resection rates are clinically meaningful only if they translate into improved stoma-free survival and quality of life. Restorative surgery at the cost of severe low anterior resection syndrome may not confer the anticipated benefit, particularly in patients with low rectal tumours where functional sequelae are most pronounced. Future studies should therefore go beyond surgical endpoints and incorporate patient-reported outcomes encompassing bowel function and long-term stoma-free survival to assess the true clinical value of higher restorative resection rates with R-TME. Additionally, any potential benefits of R-TME must also be weighed against higher procedural costs and the limited availability of robotic systems in many healthcare settings.

Subgroup analyses suggested that the association between R-TME and restorative procedure rates was most pronounced in studies including only procedures performed beyond 2020 (OR 2.86 (95% c.i. 1.80 to 4.53), *P* < 0.001). This apparent time-related effect likely reflects the maturation of expertise with robotic platforms among surgeons and their teams, leading to improved procedural efficiency. Technological refinement of robotic platforms and instrumentation over the last years, alongside growing familiarity with robotic-specific techniques relevant to sphincter preservation, and improvement in patient and tumour selection as institutional experience accumulated, may have further contributed independently. As experience with R-TME continues to accumulate and robotic technology further evolves, outcomes may continue to improve. Subgroup analysis based on surgeon experience was not feasible, however, due to heterogeneous and frequently incomplete reporting, underscoring the need for more standardized reporting of learning curve metrics in future studies. The substantial between-study heterogeneity observed (*I*^2^ = 77.3%) is further reflected in the wide 95% prediction interval for the primary outcome (OR 0.42–4.85), indicating that, while the average effect favours R-TME, the magnitude of benefit is not expected to be uniform across all clinical settings.

In addition to calendar time, factors were examined that are known to increase technical complexity in rectal cancer surgery, namely male sex, associated with a narrower pelvis, and the use of NAT^39^. The pooled analyses did not demonstrate statistically significant subgroup differences when stratifying by the proportion of male patients or use of NAT. Further stratification according to the specific NAT regimen was not feasible given the aggregate study-level design and heterogeneous reporting and treatment regimens across the included studies. These subgroup analyses may have been underpowered to detect modest differences once stratified by these variables. Furthermore, the decision to perform a primary anastomosis after NAT may be influenced by similar considerations in both the robotic and laparoscopic setting, thereby attenuating potential differences between techniques.

With regards to secondary outcomes, R-TME was associated with a substantially lower conversion rate compared with L-TME (OR 0.37 (95% c.i. 0.28 to 0.49), *P* < 0.001), a finding consistent with previous reports^5^. This advantage is plausibly related to the enhanced dexterity and three-dimensional visualization offered by the robotic platform, the same technical features that may contribute to higher restorative surgery rates. Operating time was significantly longer in the robotic group (MD 36.03 (95% c.i. 8.93 to 63.13) min, *P* < 0.009). The limited number of studies reporting operating time and the variability in case complexity may have contributed to high heterogeneity amongst studies. While longer operating times are frequently cited as a drawback of robotic surgery, their clinical relevance is limited in the absence of increased morbidity. Furthermore, operating time and costs are known to decrease as surgeon experience and institutional familiarity with robotic platforms grow^40,41^. Subgroup analyses for operating time by study interval were not feasible due to the limited number of studies reporting mean operating time. Finally, no significant differences were observed between R-TME and L-TME with regard to AL (OR 0.72 (95% c.i. 0.45 to 1.15), *P* = 0.166), overall postoperative complications (OR 0.94 (95% c.i. 0.82 to 1.08), *P* = 0.408), reoperations (OR 0.93 (95% c.i. 0.74 to 1.16), *P* = 0.508), or readmissions (OR 0.76 (95% c.i. 0.48 to 1.20), *P* = 0.247). These findings support the notion that the higher restorative surgery rate with R-TME does not come at the expense of increased postoperative morbidity. However, the interpretation of AL outcomes is constrained by the absence of a standardized definition across studies. Only 3 of the 17 studies that reported this outcome specified the definition used and the three definitions were heterogeneous, encompassing clinical and radiological criteria. This variability in definition might have introduced misclassification bias, potentially leading to either underestimation or overestimation of AL rates. Centres relying solely on clinical criteria may have captured only symptomatic leaks, while those incorporating routine radiological screening may have identified subclinical cases that would otherwise go unreported. As a result, the pooled estimate for AL should be interpreted with caution.

The present findings should also be interpreted within the broader landscape of minimally invasive rectal cancer surgery, which encompasses not only R-TME and L-TME but also transanal total mesorectal excision (TaTME). TaTME offers a complementary bottom-up approach that may be particularly advantageous for low rectal tumours in anatomically challenging patients. A recent systematic review and meta-analysis by Chaouch *et al*.^42^ directly comparing R-TME with TaTME found no significant differences in postoperative morbidity, conversion rate, or oncological outcomes, with the exception of a higher rate of complete TME in the robot-assisted group. The clinical relevance of higher anastomosis rates with both R-TME and TaTME compared with L-TME is supported by a retrospective cohort study by Burghgraef *et al*.^43^, which demonstrated that both techniques were associated with a significantly higher primary anastomosis rate compared with L-TME. Their findings suggest that higher primary anastomosis rates with R-TME and TaTME translate into a meaningful long-term benefit, being stoma-free survival. Future research directly comparing all three minimally invasive approaches, with restorative surgery, permanent stoma rate, and quality of life as predefined outcomes, would help clarify the relative advantages of each technique across different patient profiles.

This study has several strengths. By pooling data from >7000 patients, this analysis provides substantial statistical power and enhances the generalizability of the findings. The inclusion of both randomized and prospective cohort studies reflects contemporary real-world practice. In addition, pre-planned subgroup analyses, including study interval, sex distribution, and use of NAT, allowed exploration of potential sources of heterogeneity and offered nuance beyond the primary pooled estimate.

Several limitations should also be acknowledged. First, there was considerable clinical and methodological heterogeneity among the included studies, particularly in the reporting of tumour height and other anatomical details. As these variables are strong determinants of whether a restorative procedure is technically feasible, their incomplete reporting represents a key limitation of the current evidence base, precluding subgroup analyses by tumour height and leaving open the possibility that differences in case selection between R-TME and L-TME centres confounded the observed association. Standardized reporting of tumour height, i.e. distance to the anorectal junction, should be considered a minimum requirement in future comparative studies of minimally invasive rectal cancer surgery. Second, none of the included studies predefined restorative surgery as their primary endpoint; consequently, the conclusions are largely based on secondary or exploratory analyses. Third, residual confounding related to centre-level factors may have influenced the observed associations, as centres adopting robotic platforms may be characterized by higher institutional volumes, greater subspecialization, and a stronger emphasis on sphincter-preserving surgery. Furthermore, surgeon experience was not consistently or uniformly reported across studies, which may have introduced additional confounding and variability in the observed outcomes. Finally, the evidence base is dominated by non-randomized studies, which are prone to residual and unmeasured confounding and selection bias. In some centres, R-TME may have been preferentially offered for more complex or lower rectal tumours, potentially biasing against the robotic approach, whereas, in others, patient selection may have favoured robotics for less complex cases. Although established tools (RoB 2 and MINORS) and the GRADE framework were used to appraise the quality and certainty of the evidence, the overall certainty for the primary outcome is best interpreted as moderate.

In conclusion, this systematic review and meta-analysis, to the authors’ knowledge the first to specifically focus on restorative surgery rates after minimally invasive TME for rectal cancer, demonstrates that R-TME is associated with a higher likelihood of restorative surgery without an increase in postoperative morbidity. The association of robot-assisted surgery was most evident in recent studies, reflecting growing expertise. The findings also confirm that R-TME is associated with a significantly lower conversion rate compared with L-TME. After an initial trajectory of scepticism and caution, as was the case for laparoscopic surgery when it arose, robot-assisted surgery seems to be surpassing its predecessor in effectiveness.

## Supplementary Material

znag106_Supplementary_Data

## Data Availability

All data underlying the results are available upon request.
