# Supplementary material for: Restorative resection in robot-assisted versus laparoscopic total mesorectal excision: systematic review and meta-analysis
Source: Br J Surg. 2026 Oct 6;113(10):znag106. doi: 10.1093/bjs/znag106 (PMC13640960; doi:10.1093/bjs/znag106)
Supplement: Supplementary file 1 [file znag106_supplementary_data.docx]

**Restorative Resection in Robot-assisted versus Laparoscopic Total Mesorectal Excision: a Systematic Review and Meta-Analysis.**

Authors: Charlotte M.S. Genders, MD^1,2*^, Jory Wentink, MD^1,2*^, Ritch T.J. Geitenbeek, MD, PhD^1,2^, Roel Hompes, MD, PhD^3,4^, Jasper B. van Praagh, MD, PhD^1,5^, Prof. Esther C.J. Consten, MD, PhD^1,2^

^1^University Medical Centre Groningen, Department of Surgery, Groningen, the Netherlands

^2^Meander Medical Centre, Department of Surgery, Amersfoort, the Netherlands

^3^Amsterdam University Medical Centre, Department of Surgery, Amsterdam, the Netherlands.

^4^Cancer Treatment and Quality of Life, Cancer Centre Amsterdam, Amsterdam, the Netherlands.

^5^Deventer Hospital, Department of Surgery, Deventer, the Netherlands

* Both authors share first authorship

**Corresponding author.**

C.M.S. Genders

University Medical Centre Groningen, Department of Surgery

Hanzeplein 1, 9713 GZ Groningen, the Netherlands

E-mail: [c.m.s.genders@umcg.nl](mailto:c.m.s.genders@umcg.nl)

**Supplementary Materials - Index**

| **Supplementary Appendixes** |  |
| --- | --- |
| Supplementary 1: Search Syntax | *pag. 2* |
| **Supplementary Figures and Tables** |  |
| Supplementary 2: Overview of patient demographics in included studies | *pag. 4* |
| Supplementary 3: Overview of perioperative outcomes in included studies  Supplementary 4: Overview of postoperative outcomes in included studies  Supplementary 5: Quality assessment of included randomised controlled trials  Supplementary 6: Quality assessment of included non-randomised studies  Supplementary 7: Funnel Plots  Supplementary 8: Forest plots | *pag. 6*  *pag. 7*  *pag. 9*  *pag. 10*  *pag. 11*  *pag. 12* |
|  |  |
|  |  |

**Supplementary Appendixes**

**Appendix 1: Search Syntax**

*Pubmed*

("Rectal Neoplasms"[Mesh] OR ((rect*[tiab]) AND (cancer*[tiab] OR neoplasm*[tiab] OR tumo*[tiab] OR carcinoma[tiab])))

AND

(((total mesorect*[tiab]) AND (excision*[tiab] OR removal*[tiab] OR surg*[tiab]))

OR TME[tiab])

AND

(“Laparoscopy”[Mesh] OR laparo*[tiab] OR “Robotics”[Mesh] OR robot*[tiab] OR “minimally invasive”[tiab] OR MIS[tiab])

*Embase*

('rectum tumor'/exp OR (rect*:ab,ti,kw AND (cancer* OR neoplasm* OR tumo* OR carcinoma*):ab,ti,kw))

AND

((total mesorect*:ab,ti,kw AND (excision* OR removal* OR surg*):ab,ti,kw) OR TME:ab,ti,kw)

AND

('laparoscopy'/exp OR laparo*:ab,ti,kw OR 'robotics'/exp OR robot*:ab,ti,kw OR ‘minimally invasive surgery’:ab,ti,kw OR MIS:ab,ti,kw)

*Web of science*

TS=((("total mesorect*") AND (excision* OR removal* OR surg*)) OR TME)

AND

TS=(laparo* OR robot* OR "minimally invasive" OR MIS)

AND

TS=((rect*) AND (cancer* OR neoplasm* OR tumo* OR carcinoma))

*Cochrane Library*

([mh "Rectal Neoplasms"] OR ((rect*:ti,ab,kw) AND (cancer*:ti,ab,kw OR neoplasm*:ti,ab,kw OR tumo*:ti,ab,kw OR carcinoma*:ti,ab,kw)))

AND

(((("total" NEXT mesorect*):ti,ab,kw) AND (excision*:ti,ab,kw OR removal*:ti,ab,kw OR surg*:ti,ab,kw))

OR TME:ti,ab,kw)

AND

([mh Laparoscopy] OR laparo*:ti,ab,kw OR [mh Robotics] OR robot*:ti,ab,kw OR "minimally invasive":ti,ab,kw OR MIS:ti,ab,kw)

**Supplementary Figures and Tables**

**Supplemetary Table 2.** Overview of patient demographics

| Study |  | Surgeon experience | Age | Sex, no. female | BMI | ASA score | | | | Tumour height, cm | Neoadjuvant treatment |
| --- | --- | --- | --- | --- | --- | --- | --- | --- | --- | --- | --- |
|  |  |  |  |  |  | I | II | III | IV |  |  |
| Bianchi et al.^18^ | *R-TME* | >2 procedures | 69 [33-83]ᵇ | 7 | 24.6 [18.5-31.2]ᵇ | Unknown |  |  |  | Unknown | 13 (52) |
|  | *L-TME* | >100 procedures | 62 [42-77]ᵇ | 8 | 26.5 [18.1-35]ᵇ | Unknown |  |  |  | Unknown | 10 (40) |
| Kim et al. ^19^ | *R-TME* | Not specified | 54.13 (8.52)ᵃ | 12 (40) | 24.36 (2.44) | 29 (96.7) | 1 (3.3) | 0 | 0 | <6: 6 (20) | 10 (33.3) |
|  | *L-TME* | Not specified | 56.85 (11.14)ᵃ | 19 (48.7) | 24.01 (2.19) | 39 (100) | 0 | 0 | 0 | <6: 8 (20.5) | 12 (30.8) |
| Park et al. ^20^ | *R-TME* | Not specified | <60: 14 (43.8) | 0 | 23.8 (2.3) | Unknown |  |  |  | <7: 19 (59.4) | 15 (46.9) |
|  | *L-TME* | Not specified | <60: 15 (46.9) | 0 | 23.6 (2.7) | Unknown |  |  |  | <7: 14 (43.8) | 9 (28.1) |
| Levic et al. ^21^ | *R-TME* | Not specified | 65 [23-83]ᵇ | 22 (39.3) | 24.8 [16-34.5]ᵇ | 17 (30.4) | 35 (62.5) | 4 (7.1) | 0 | 8 [1-15]ᵇ | Unknown |
|  | *L-TME* | Not specified | 69 [49-87]ᵇ | 19 (52.8) | 23.8 [16-32]ᵇ | 9 (25) | 22 (61.1) | 5 (13.9) | 0 | 8 [2-15]ᵇ | Unknown |
| Alleman et al. ^22^ | *R-TME* | >50 procedures | 64 (12) | 8 (40) | 25.9 (9) | 5 (25) | 9 (45) | 6 (30) | 0 | 4.1 (1.7)ᵃ | CRT: 13 (65) |
|  | *L-TME* | Not specified | 65 (13) | 16 (40) | 24.2 (7) | 3 (8) | 27 (67) | 10 (25) | 0 | 4.8 (2.6)ᵃ | CRT: 24 (60) |
| Yamaguchi et al. ^23^ | *R-TME* | Not specified | 64.8 (10.8) | 63 (31.0) | 23.4 (3.16) | 57 (28.1) | 137 (67.5) | 9 (4.4) | 0 | Unknown | CRT 1 (0.5) |
|  | *L-TME* | Not specified | 65.9 (10.8) | 85 (35.6) | 23.1 (3.64) | 68 (28.5) | 153 (64.0) | 18 (7.5) | 0 | Unknown | CRT 0 |
| Ahmed et al. ^24^ | *R-TME* | Not speified | 69 [63-75]ᵇ | 28 (28.3) | 27 [24-30]ᵇ | Unknown |  |  |  | 7 [5-10]ᵇ | Rtx: 27 (27.3) |
|  | *L-TME* | >500 procedures | 68 [62-74]ᵇ | 27 (31.8) | 27 [24-31]ᵇ | Unknown |  |  |  | 7 [5-10]ᵇ | Rtx: 9 (22.4) |
| Jayne et al. ^25^ | *R-TME* | >10 procedures | 64.4 (10.98)ᵃ | 76 (32.1) | 0-24.9: 93 (39.2); 25.0-29.9: 90 (38.0); ≥30.0: 54 (22.8) | 39 (16.5) | 150 (63.3) | 47 (19.8) | 1 (0.4) | 0-5: 57 (24.2) | 111 (46.8) |
|  | *L-TME* | >10 procedures | 65.5 (11.93)ᵃ | 75 (32.1) | 0-24.9: 87 (37.2); 25.0-29.9: 92 (39.3); ≥30.0: 55 23.5) | 52 (22.2) | 124 (53.0) | 52 (22.2) | 0 | 0-5: 61 (26.5) | 108 (46.2) |
| Law et al. ^26^ | *R-TME* | Not specified | 65 [34-90]ᵇ | 72 (32.7) | 24.9 | 26 (11.8) | 147 (66.8) | 47 (21.4) | 0 | 7.0 [0-12]ᵇ | Rtx: 91 (41.1) |
|  | *L-TME* | > 250 procedures | 67 [23-96]ᵇ | 74 (43.3) | 24.6 | 20 (11.7) | 103 (60.2) | 35 (20.5) | 0 | 8.0 [0-12]ᵇ | Rtx: 50 (29.2) |
| Panteleimonitis et al. ^27^ | *R-TME* | Not specified | 69 [64-74.75]ᵇ | 13 (27.1) | Male 27 [25–28.25], female 27 [24.25–29.5]ᵇ | 4 (9) | 39 (85) | 3 (7) | 0 | 7.91 (0.54)ᵃ | Rtx: 23 (48), Ctx: 11 (23) |
|  | *L-TME* | Not specified | 70 [63-75.25]ᵇ | 29 (37.2) | Male 26 [23–29.5], female 26 [24–32.5]ᵇ | 12 (15) | 51 (65) | 15 (19) | 0 | 9.36 (0.38)ᵃ | Rtx: 28 (36), Ctx: 7 (9) |
| Crolla et al. ^28^ | *R-TME* | Not specified | 67 (9.64)ᵃ | 81 (44.0) | 26.4 (3.86)ᵃ | 27 (16.1) | 116 (69.0) | 25 (14.9) | 0 | Unknown | Rtx: 98 (58.3), Ctx: 46 (27.4) |
|  | *L-TME* | Not specified | 68.1 (10.7)ᵃ | 55 (32.7) | 25.8 (3.90)ᵃ | 34 (18.5) | 104 (56.5) | 46 (24.5) | 1 (0.5) | Unknown | Rtx: 92 (50.3), Ctx: 23 (12.7) |
| Kim et al. ^29^ | *R-TME* | >30 procedures | 60.4 (9.7)ᵃ | 15 (22.7) | 24.1 (3.3)ᵃ | 20 (30.3) | 46 (69.7) | 0 | 0 | ≤5: 33 (50) | CRT: 51 (77.3) |
|  | *L-TME* | >500 procedures | 59.7 (11.7)ᵃ | 21 (28.8) | 23.6 (3.0)ᵃ | 30 (41.1) | 43 (58.9) | 0 | 0 | ≤5: 35 (48) | CRT: 58 (79.5) |
| Megevand et al. ^30^ | *R-TME* | Not specified | 70 | 12 (34.3) | 24.6 | 6 (17.1) | 22 (62.9) | 7 (20.0) | 0 | 8 | CRT: 11 (31.4) |
|  | *L-TME* | Not specified | 66 | 17 (48.6) | 25 | 5 (14.3) | 24 (68.6) | 9 (17.1) | 0 | 8 | CRT: 4 (11.4) |
| Tolstrup et al. ^31^ | *R-TME* | >10 procedures | 63 (10.9)ᵃ | 7 (28) | 27 (4.5)ᵃ | 11 (44) | 7 (28) | 0 | 0 | Unknown | Unknown |
|  | *L-TME* | >10 procedures | 68 (9.9)ᵃ | 6 (23) | 28 (4.3)ᵃ | 10 (38.5) | 8 (30.8) | 1 (3.8) | 0 | Unknown | Unknown |
| Feng et al. ^4^ | *R-TME* | >50 procedures | 59.1 (11.0)ᵃ | 230 (39.2) | 23.5 (3.3)ᵃ | 324 (55.3) | 230 (39.2) | 32 (5.5) | 0 | 5.9 (2.4)ᵃ | 254 (43.3) |
|  | *L-TME* | >50 procedures | 60.7 (9.8)ᵃ | 231 (39.5) | 23.5 (3.1)ᵃ | 318 (54.4) | 240 (41.0) | 27 (4.6) | 0 | 5.8 (2.6)ᵃ | 257 (43.9) |
| Liu et al. ^32^ | *R-TME* | >65 procedures | 58.7 (7.34)ᵃ | 38 (42.2) | 22.6 (1.66)ᵃ | 53 (58.9) | 35 (38.9) | 2 (2.2) | 0 | 5.17 (0.74)ᵃ | 50 (55.6) |
|  | *L-TME* | >200 procedures | 58.53 (8.78)ᵃ | 20 (44.4) | 23.3 (2.4)ᵃ | 27 (60) | 18 (40) | 0 | 0 | 5.23 (0.92)ᵃ | 27 (60) |
| Myrseth et al. ^33^ | *R-TME* | Not specified | 69 [60-75]ᵇ | 144 (38) | <18.5 6 (1.7); 18.8–25 131 (36.1); 25–30 146 (40.2); >30 80 (22.0) | I-II: 231 (61.6) |  | III-IV: 144 (38.4) |  | <5: 85 (35.0) | 143 (38.1) |
|  | *L-TME* | Not specified | 69 [60-76]ᵇ | 358 (39) | <18.5 18 (2); 18.8–25 380 (42.4); 25–30 350 (39.1); >30 148 (16.5) | I-II: 640 (70.4) |  | III-IV: 269 (29.6) |  | <5: 159 (26.6) | 323 (25.5) |
| Duhoky et al. ^7^ | *R-TME* | >35 procedures | 68.00 [61.00-75.00]^b^ | 385.4 (36.4)^a^ | 25.95 [23.45-28.76]ᵇ | I-II: 859.5 (81.3) |  | III-IV: 198.0 (18.7) |  | 6.80 [4.50-9.00]ᵇ | 565.5 (53.5) |
|  | *L-TME* | >35 procedures | 68.00 (60.00-[75.00]^b^ | 249.4 (36.6)^a^ | 25.74 [23.14-28.92]ᵇ | I-II: 553.6 (81.3) |  | III-IV: 127.3 (18.7) |  | 6.50 [4.98-9.00]ᵇ | 356.0 (52.3) |
| Guerrero-Ortiz et al. ^34^ | *R-TME* | Not specified | 65.82 (13.06)ᵃ | 38 (40) | 25.63 (3.82)ᵃ | I-II: 68 (71.6) |  | III-IV: 27 (28.4) |  | 4.04 (1.76)ᵃ | 63 (72.4) |
|  | *L-TME* | Not specified | 68.43 (10.52)ᵃ | 33 (37.9) | 26.51 (3.66)ᵃ | I-II:  59 (67.8) |  | III-IV:  28 (32.2) |  | 4.20 (2.09)ᵃ | 58 (61.1) |
| Kitaguchi et al. ^35^ | *R-TME* | Not specified | 63 [46-78]ᵇ | 7 (33) | 22.5 [20.0-32.7]ᵇ | 2 (10) | 17 (81) | 2 (10) | 0 | 5.3 [0-10]ᵇ | CRT: 12 (57) |
|  | *L-TME* | Not specified | 63 [41–77]ᵇ | 11 (27) | 23.7 [16.8-30.3]ᵇ | 3 (7) | 32 (78) | 6 (15) | 0 | 5.5 [0-9]ᵇ | Not specified |

ᵃ Mean (SD)

ᵇ Median [Q1-Q3]

*ASA,* American Society of Anesthesiologists; *BMI,* body mass index; *CRT,* chemoradiotherapy; *Ctx,* chemotherapy; *L-TME,* laparoscopic total mesorectal excision; *no,* number; *R-TME,* robot-assisted total mesorectal excision; *Rtx*, radiotherapy.

**Supplementary Table 3.** Overview of perioperative outcomes

| Study |  | Operative time | Estimated Blood loss | Conversion, n (%) | Defunctioning stoma, n (%) |
| --- | --- | --- | --- | --- | --- |
| Bianchi et al.^18^ | *R-TME* | Unknown | Unknown | 0 | 10 (40) |
|  | *L-TME* | Unknown | Unknown | 1 (4) | 5 (20) |
| Kim et al. ^19^ | *R-TME* | Unknown | Unknown | Unknown | Unknown |
|  | *L-TME* | Unknown | Unknown | Unknown | Unknown |
| Park et al. ^20^ | *R-TME* | Unknown | Unknown | Unknown | Unknown |
|  | *L-TME* | Unknown | Unknown | Unknown | Unknown |
| Levic et al. ^21^ | *R-TME* | 247 [130-511]ᵇ | 50 [0-400]ᵇ | 3 (5.4) | Unknown |
|  | *L-TME* | 295 [108-465]ᵇ | 35 [0-400]ᵇ | 0 | Unknown |
| Alleman et al. ^22^ | *R-TME* | 291 | 58 (76)ᵃ | 1 (5) | Unknown |
|  | *L-TME* | 313 | 219 (421)ᵃ | 8 (20) | Unknown |
| Yamaguchi et al. ^23^ | *R-TME* | 232.9 (72.0)ᵃ | 15.4 (26.4)ᵃ | 0 | 17 (10.1) |
|  | *L-TME* | 227.6 (62.6)ᵃ | 39.1 (85.1)ᵃ | 8 (3.3) | 13 (6.7) |
| Ahmed et al. ^24^ | *R-TME* | 240 [205-290]ᵇ | 10 [0-12.5]ᵇ | 0 | Unknown |
|  | *L-TME* | 270 [220-330]ᵇ | 100 [10-212.5]ᵇ | 4 (4.7) | Unknown |
| Jayne et al. ^25^ | *R-TME* | 298.5 (88.71)ᵃ | Unknown | 19 (8.1) | 142 (75.9) |
|  | *L-TME* | 261.0 (83.24)ᵃ | Unknown | 28 (12.2) | 157 (85.3) |
| Law et al. ^26^ | *R-TME* | 260 [137–671]ᵇ | 100 [10–2500]ᵇ | 2 (0.8) | 164 (79.6) |
|  | *L-TME* | 225 [101–520]ᵇ | 100 [10–2000]ᵇ | 6 (3.5) | 115 (75.7) |
| Panteleimonitis et al. ^27^ | *R-TME* | Unknown | Unknown | Unknown | Unknown |
|  | *L-TME* | Unknown | Unknown | Unknown | Unknown |
| Crolla et al. ^28^ | *R-TME* | 219 (47.7)ᵃ | Unknown | 3 (1.8) | 71 (63.4) |
|  | *L-TME* | 172 (48.4)ᵃ | Unknown | 23 (12.5) | 78 (63.9) |
| Kim et al. ^29^ | *R-TME* | 339.2 (80.1)ᵃ | 100 [range 0–1000] | 1 (1.5) | Unknown |
|  | *L-TME* | 227.8 (65.6)ᵃ | 50 [range 0–300] | 0 | Unknown |
| Megevand et al. ^30^ | *R-TME* | 252.5 [ 214-300]ᵇ | Unknown | 0 | Unknown |
|  | *L-TME* | 225 [194-255]ᵇ | Unknown | 8 (23) | Unknown |
| Tolstrup et al. ^31^ | *R-TME* | Unknown | 152 (43)ᵃ | 1 (4) | Unknown |
|  | *L-TME* | Unknown | 170 (57)ᵃ | 10 (38.5) | Unknown |
| Feng et al. ^4^ | *R-TME* | 173 [140- 225]ᵇ | 40 [30-100]ᵇ | 10 (1.7) | 129 (26.5) |
|  | *L-TME* | 170 [140-209]ᵇ | 50 [40-100]ᵇ | 23 (3.9) | 117 (26.1) |
| Liu et al. ^32^ | *R-TME* | 232.5 (75.9)ᵃ | 65.9 (35.3)ᵃ | 2 (2.2) | 70 (80.5) |
|  | *L-TME* | 205.5 (85.0)ᵃ | 95.1 (78.9)ᵃ | 3 (6.8) | 35 (97.2) |
| Myrseth et al. ^33^ | *R-TME* | Unknown | Unknown | 8 (2.1) | Unknown |
|  | *L-TME* | Unknown | Unknown | 87 (9.6) | Unknown |
| Duhoky et al. ^7^ | *R-TME* | 220.00 [164.88-270.00]ᵇ | Unknown | 37.4 (3.5) | 303.9 (31.2) |
|  | *L-TME* | 181.00 [135.00-246.00]ᵇ | Unknown | 41.7 (6.1) | 269.1 (49.3) |
| Guerrero-Ortiz et al. ^34^ | *R-TME* | 239 (76.48)ᵃ | 58.55 (51.68)ᵃ | 0 | Unknown |
|  | *L-TME* | 248.61 (73.50)ᵃ | 131.68 (191.92)ᵃ | 3 (3.4) | Unknown |
| Kitaguchi et al. ^35^ | *R-TME* | 549 [311–638]ᵇ | 70 [0-660]ᵇ | 0 | 15 (100) |
| Bianchi et al.^18^ | *L-TME* | 455 [281–640]ᵇ | 130 [0-1200]ᵇ | 0 | 27 (100) |

ᵃ Mean (SD)

ᵇ Median [Q1-Q3]

*L-TME,* laparoscopic total mesorectal excision; *R-TME,* robot-assisted total mesorectal excision.

**Supplementary Table 4.** Overview of postoperative outcomes

| Study |  | Length of stay | Reoperation, n (%) | Readmission, n (%) | Postoperative complication, n (%) | Anastomotic leakage, n (%)* | Anastomotic leakage definition |
| --- | --- | --- | --- | --- | --- | --- | --- |
| Bianchi et al.^18^ | *R-TME* | Unknown | 1 (5.6) | Unknown | 4 (16) | 1 (5.6) | Not specified |
|  | *L-TME* | Unknown | 2 (10.5) | Unknown | 5 (20) | 2 (10.5) |  |
| Kim et al. ^19^ | *R-TME* | Unknown | Unknown | Unknown | Unknown | Unknown | NA |
|  | *L-TME* | Unknown | Unknown | Unknown | Unknown | Unknown |  |
| Park et al. ^20^ | *R-TME* | Unknown | Unknown | Unknown | Unknown | 3 (9.7) | Not specified |
|  | *L-TME* | Unknown | Unknown | Unknown | Unknown | 3 (9.1) |  |
| Levic et al. ^21^ | *R-TME* | 8 [4-100]ᵇ | Unknown | 13 (23.2) | 12 (23.2) | 6 (18.8) | Not specified |
|  | *L-TME* | 7 [3-51]ᵇ | Unknown | 5 (13.9) | 10 (27.8) | 3 (15.0) |  |
| Alleman et al. ^22^ | *R-TME* | Unknown | 3 (15) | 1 (5) | 8 (40) | CD 3-4: 0 | Not specified |
|  | *L-TME* | Unknown | 5 (13) | 5 (12) | 14 (35) | CD 3-4: 2 (5.9) |  |
| Yamaguchi et al. ^23^ | *R-TME* | 7.3 (2.3)ᵃ | Unknown | Unknown | Unknown | 3 (1.8) | *“Confirmed by contrast radiography and computed tomography”* |
|  | *L-TME* | 9.3 (6.7)ᵃ | Unknown | Unknown | Unknown | 7 (3.6) |  |
| Ahmed et al. ^24^ | *R-TME* | 7 [5-13]ᵇ | 4 (4.0) | 9 (9.1) | Unknown | 1 (1.2) | Not specified |
|  | *L-TME* | 9 [7-15]ᵇ | 4 (4.7) | 10 (11.8) | Unknown | 2 (2.3) |  |
| Jayne et al. ^25^ | *R-TME* | 8.0 (5.85)ᵃ | Unknown | Unknown | 78 (33.1) | 22 (12.2) | Not specified |
|  | *L-TME* | 8.2 (6.03)ᵃ | Unknown | Unknown | 73 (31.7) | 18 (9.9) |  |
| Law et al. ^26^ | *R-TME* | 6 [2-64]ᵇ | 7 (3.2) | Unknown | 42 (19.1) | 11 (5.3) | ^c^ |
|  | *L-TME* | 6 [2-83]ᵇ | 4 (2.3) | Unknown | 38 (22.2) | 3 (2.0) |  |
| Panteleimonitis et al. ^27^ | *R-TME* | Unknown | Unknown | Unknown | Unknown | 4 (10) | Not specified |
|  | *L-TME* | Unknown | Unknown | Unknown | Unknown | 3 (5) |  |
| Crolla et al. ^28^ | *R-TME* | 6 [2-67]ᵇ | Unknown | Unknown | Unknown | 5 (4.5) | *“Radiologic or operative sign of a defect of the anastomosis, including deep abscesses next to the anastomosis”* |
|  | *L-TME* | 7 [3-104]ᵇ | Unknown | Unknown | Unknown | 10 (8.2) |  |
| Kim et al. ^29^ | *R-TME* | 10.3 (3.4)ᵃ | 2 (3.0) | Unknown | 23 (34.8) | 8 (12.3) | Not specified |
|  | *L-TME* | 10.9 (7.4)ᵃ | 2 (2.7) | Unknown | 17 (23.3) | 5 (7.1) |  |
| Megevand et al. ^30^ | *R-TME* | Unknown | 3 (10.7) | 0 | CD 3-5: 6 (17.1) | 1 (3.6) | Not specified |
|  | *L-TME* | Unknown | 3 (10.7) | 0 | CD 3-5: 7 (20.0) | 4 (13.3) |  |
| Tolstrup et al. ^31^ | *R-TME* | 8.9 (5.6)ᵃ | Unknown | Unknown | 10 (40) | Unknown | NA |
|  | *L-TME* | 9.5 (7.7)ᵃ | Unknown | Unknown | 10 (38.4) | Unknown |  |
| Feng et al. ^4^ | *R-TME* | 7 [7-11]ᵇ | 14 (2.4) | 17 (2.9) | 95 (16.2) | 25 (5.1) | Not specified |
|  | *L-TME* | 8 [7-12]ᵇ | 24 (4.1) | 20 (3.4) | 135 (23.1) | 37 (8.2) |  |
| Liu et al. ^32^ | *R-TME* | 7.5 (1.2)ᵃ | Unknown | Unknown | Unknown | 3 (3.4) | Not specified |
|  | *L-TME* | 8.0 (1.4)ᵃ | Unknown | Unknown | Unknown | 5 (13.9) |  |
| Myrseth et al. ^33^ | *R-TME* | 5 [3-7]ᵇ | 35 (9.3) | Unknown | Accordion ≥3: 43 (11.5) | 14 (7.3) | Not specified |
|  | *L-TME* | 6 [4-9]ᵇ | 71 (7.8) | Unknown | Accordion ≥3: 112 (12.3) | 27 (4.9) |  |
| Duhoky et al. ^7^ | *R-TME* | 6.00 [5.00-11.00]ᵇ | 93.9 (9) | 144.8 (13.7) | 484.4 (45.8) | 116.8 (12.7) | Not specified |
|  | *L-TME* | 6.00 [4.00-10.00]ᵇ | 69.1 (10.3) | 107.4 (15.9) | 300.9 (45.0) | 59.3 (12.9) |  |
| Guerrero-Ortiz et al. ^34^ | *R-TME* | 10.08 (9.33)ᵃ | 8 (8.4) | 2 (2.1) | Unknown | 7 (8.0) | Grade A, B and C according to Rahbari et al.^d^ |
|  | *L-TME* | 8.99 (7.45)ᵃ | 8 (9.2) | 13 (15) | Unknown | 6 (6.9) |  |
| Kitaguchi et al. ^35^ | *R-TME* | 15 [9-22]ᵇ | 0 | Unknown | CD ≥2: 4 (19) | 1 (6.7) | Not specified |
|  | *L-TME* | 22 [10-60]ᵇ | 1 (2) | Unknown | CD ≥2: 20 (49) | 5 (18.5) |  |

ᵃ Mean (SD)

ᵇ Median [Q1-Q3]

^c^ Clinical anastomotic leak was considered to be present if any of the following features was observed: the presence of peritonitis caused by anastomotic dehiscence, the presence of feculent substances and gas from the pelvic drain or the presence of a pelvic abscess with demonstration of anastomotic leak by rectal examination, sigmoidoscopy or contrast study

^d^ Rahbari N.N., Weitz J., Hohenberger W., et al.: Definition and grading of anastomotic leakage following anterior resection of the rectum: a proposal by the International Study Group of Rectal Cancer . Surgery 2010; 147: pp. 339-351.

* Percentage of patients with anastomotic leakage of total number of restorative procedures

*CD,* Clavien-Dindo classification*; L-TME,* laparoscopic total mesorectal excision; *n,* number; *R-TME,* robot-assisted total mesorectal excision.

**Supplementary Figure 5.** Quality assessment of included randomised controlled trials using the Cochrane Risk of Bias (RoB 2) Tool

**Supplementary figure 6.** Quality assessment of included non-randomised studies using the Methodological Index for Non-Randomised Studies (MINORS) Tool

**Supplementary figure 7.** Funnel plot for (A) restorative surgery and (B) anastomotic leakage.

**Supplementary figure 8.** Forest plots of pooled odds ratios with 95% confidence intervals for subgroup analysis of (A) Study size, (B) Study design, (C) Neoadjuvant therapy use* and (D) Male gender**, and (E) ASA classification***.

*CI,* confidence interval; *L-TME,* laparoscopic total mesorectal excision*; NATuse, neoadjuvant therapy use, OR,* odds ratio; *R-TME, R-TME-assisted total mesorectal excision.*

** Neoadjuvant therapy use was categorised as High (≥70%), Moderate (30-69%), and Low (≤30%)*

*** Proportion of male participants was categorised as High (≥67%) and Low (<67%)*

**** Proportion of ASA III-IV participants was categorised as High (≥25%) and Low (<25%)*
